# The Medical Student's Vade Mecum

**Published:** 1894-09-22

**Authors:** 


### The Medical Student's Vade Mecum.

### THE METROPOLITAN MEDICAL SCHOOLS.

### Some Results of a Personal Inquiry.

The first requisite for the training of a doctor is sufficient opportunity for the study of disease; and where should these opportunities be more abundant than in London, the largest city in the world? It may be that the system of isolated hospitals, despite its many advantages, is not the best from the educational point of view; and it is no doubt true that more might be made of the unrivalled opportunities for clinical study which London offers; but when all has been said, the place of London in the world of medicine is one of the highest, as undoubtedly London is the chief centre of medical education in this country.

Not very long ago the metropolis to all intents and purposes trained doctors for all England. Her only rival was Edinburgh. But of late years medical schools have been springing up in all the large provincial cities. Bristol, Manchester, Liverpool, Birmingham, Leeds, Newcastle, and other towns have now their well-equipped medical schools. Cambridge has established a full course in medicine which has been very successful, and Oxford has recently followed suit. But for all this there has been no appreciable falling off in the number of students that come to London. Even of those who take their science classes in the provinces—and in many of the provincial schools the science teaching is admirable—a large proportion find their way in the long run to one or other of the metropolitan hospitals.

And yet the path of the parent who has satisfied himself that the best thing he can do for his boy destined for medicine is to send him to one of the London schools is often dark enough. That a right-minded parent should hesitate about exposing his boy to the dangers and the temptations of this modern Babylon is but natural; but on one point let him be reassured. Student life in London to-day is not what it was fifty, thirty, or even twenty years ago. Dickens', Thackeray's, Albert Smith's medical student has disappeared; and his successor of to-day is a well-behaved gentlemanly fellow, who has realised somewhat of the dignity of the profession he has adopted, and whom the exigencies of the five years' curriculum have left no time for irresponsible frivolities, not to speak of dissipation. To say that there are some dishonourable exceptions to this rule is only to say that among medical students there are men of weak moral fibre as there are in every class of society. And this has to be said for the authorities of the London medical schools, that one and all they do their best to see that their men work and conduct themselves as gentlemen. Student life in London has its temptations still, but these are not sensibly greater than in many other walks of life.

Having conquered his anxiety on this score, the parent is confronted with the question of which of the eleven metropolitan schools is to have the training of his son. It is not easy to make the choice, for each one of the eleven is of good repute.

It was in order to obtain information as to those things that are of most concern to beginners in medicine and their parents that a representative of The Hospital set forth to visit the several London medical schools and to interview, as far as that was feasible, deans, wardens, and others closely associated with the work of each. It has here to be acknowledged that without exception information was most courteously granted, and that, whether the object in view has or has not been attained, the expedition was a very pleasant one.

Keeping in view the case of the anxious and perplexed parent in the country our representative made special inquiries as to the manner of life of the medical student, and as to the system of discipline that obtains. Under these heads the replies elicited were very satisfactory. There is firm and yet kindly treatment throughout. Not only the intellectual but the moral well-being of the student is the subject of care. There are medical schools of which it has been said that the authorities seemed to have discharged their responsibility towards the student when they had taken his fees. There is not one of the London schools of which this can be said. It was pleasant indeed to hear the kindly, one had almost said the affectionate, tones in which the deans spoke of their men, and to learn of the care and wisdom and sympathy exerted on behalf of the student at every stage of his career.

### The London Hospital College.

As there was a certain convenience in working from east to west, it seemed good to make a beginning with the medical school attached to the great London Hospital in the Whitechapel Road. Everybody who knows anything about hospitals knows that the largest in this country is the London. It contains 776 beds, and it is the only general hospital in the densely peopled world of East London. The bearing of this fact upon the rank of its medical school as a clinical school is obvious. There is no hospital in this country which affords better opportunities for the practical study of medical and surgical diseases in all their variety. Here is one fact that illustrates what these opportunities are: The number of major operations performed at the hospital during the year 1893 was 1,575—an average of over four per day. Notwithstanding that it is so far east, the site of school and hospital is really an excellent one—airy and central, and within convenient access by train of all parts of London. The history of this school, which may be said to date from 1785, is interesting and creditable, showing sure and steady progress. A great deal of thorough work has been quietly done, and a reputation has been built up which is sound, and promises to be lasting. And for much of what has been done credit is due to the late Sir Andrew Clark, who gave this school liberally of his time and of his best work. The present school buildings, adjoining the hospital, which were opened in 1887, are in structural arrangement all that could be desired, and provide ample accommodation for lecture-room, laboratory, and museum—the library is a very fine room, large and lofty. The laboratories are well equipped, and the museums are exceptionally rich in specimens. A full course of instruction, including special classes for the examinations for the University of London degrees, is provided, and the staff of teachers is an excellent one. The latest new feature in connection with the course of study is the appointment of a biologist and of a physiologist, who give instruction to those who mean to proceed to the University of London examinations, and this will doubtless encourage a larger number of men to take these higher degrees. The arrangements for clinical instruction at the hospital are most complete; they enable every student to have ample experience in dressing and clerking, and to have personal instruction from members of the hospital staff. Moreover, as might be expected, the number of resident appointments—which, it should be noted, are open without fee—is very large. In short, the "London" student decidedly reaps the benefit of his connection with a magnificent hospital. The medical school is under the direction of a College Board, composed of six members of the school staff and six of the House Committee of the hospital. The warden of the college, who is also secretary of the College Board, is Mr. Munro Scott. Mr. Munro Scott gives his whole time to the medical school and the students. He is a gentleman of genial bearing and energetic disposition, who is full of zeal for the school, and who maintains a watchful and yet kindly interest in the welfare of all the students. There is no residential college at the London Hospital; but it should be noted (1) that several members of the staff and several medical men residing in the neighbourhood take in student boarders; and (2) that a register of approved lodgings is kept by the warden. There is a dining and refreshment room within the buildings, which is a matter of great convenience to those who come a long distance, for *recherché* restaurants are not too plentiful in Whitechapel. In addition to the lecturers, there is a staff of tutors who give personal instruction, and these take the student in hand from the outset. These tutors who hold regular and frequent written and oral examinations, report regularly on the work and progress of the men whom they have in charge. The attendance, at classes is carefully registered. Moreover, the teachers make frequent reports to the College Board, and the discipline of this body is invoked in any case of serious misconduct. While everything is done to encourage work, much is also done to promote social life and healthy recreation. The various athletic clubs and societies have been amalgamated into a clubs' union. A recreation park has been leased at Lower Edmonton, which was opened last year.

### Guy's.

Let us now go from Whitechapel to the Borough to Guy's Hospital, within a few minutes' walk of London Bridge. There is not one of the hospitals of London so well known by name to the British public as Guy's, and for the reason, no doubt, that not only has Guy's a history of its own, but it has found a place in literature; the Guy's Hospital student has been a special favourite with novelists from Dickens onwards. Be this as it may, Guy's Hospital has long held a distinctive place among the medical schools of London, and its prestige increases year by year. Of this there is the best of evidence in the fact that on the list of students' entries at the London schools in October last Guy's stood highest. In what has been achieved the public spirit of those who have been unwearying in promoting the welfare of the school has had its deserved reward. What these efforts have been may easily be realised by anyone who pays a visit to the school. The buildings testify of them, especially the residential college opened in 1890, which cost £21,000, and the Petersham block, erected at a cost of £15,000, and opened last year. Guy's is the only general hospital in London which has attached to it a fully-equipped dental school. The dental department opened last year realised the highest expectations from the outset. But the success of the dental has accompanied—it has not in the least retarded—the success of the medical school. One or two facts set forth in the report for 1893-94 by Dr. Shaw, the genial and clear-headed dean of the school, who was most kind in affording information to our representative, bear this out. The report opens with the statement that during the past year the progress of the medical school has been thoroughly satisfactory. Among other things it is mentioned that in order to keep pace with the times it was necessary to increase the number of anæsthetists at the hospital, and "it is now possible to provide that every student before leaving the hospital shall have opportunity of personally administering anæsthetics under due supervision." Further on it is stated, "at the examinations of the Universities and Royal Colleges our students have fully maintained the reputation of the hospital."

These statements are not made with flourish of trumpets, but, read by those who have knowledge of the matter, they prove that the school is in a high state of efficiency. It is hardly necessary to add that teachers of skill, some of them of eminence, provide instruction in all the subjects of medical study. An hospital serving a large and thickly-peopled district, that has over 500 beds in constant occupation, provides ample opportunities for clinical study, of which the best use is made. The system of discipline that prevails, as fully explained by Dr. Shaw, speaks of a wise regard for the facts of human nature. While a severe check is put upon laziness and evil-doing, the young men have all the freedom that young men ought to have. There is a large staff of demonstrators, who make it their work to get into touch with each of the students. With these demonstrators the Dean holds regular conferences. Dr. Shaw reads over the names of the students. As each name is called the demonstrator in each of the subjects for which the young man is entered, makes his report—"all right" or otherwise. If the report is "otherwise," the Dean makes a black mark against the name. Inquiries are instituted; the delinquent is dealt with, and, if, necessary, his parents are communicated with. As a rule this brings about an amendment; but when sterner treatment is called for, reference is made to the authorities of the school, and unless the student mends his ways he becomes liable to expulsion. This, however, is rarely necessary. In connection with this subject, Dr. Shaw holds very strongly that the system of perpetual fees under which, on payment of a lump sum, the student obtains the right of attendance on lectures *ad vitam*, is not conducive to good discipline. It makes it rather a delicate matter for the authorities to rusticate, even when it becomes desirable for the sake of the school that the "weed" or the "chronic" should be expelled. The offender or his parents may turn round and say, "Now that you have got my money it is all very well for you to talk of turning me out. The fee that I paid was a perpetual fee." To be charged with breach of contract is not a pleasant thing, even when the breach of contract has been all on the side of the person who makes the charge. But incorrigibly evil in disposition must the youth be who is able to resist the wholesome influences that are brought to bear on him at Guy's. It is not too much to say that on the part of the Dean and his colleagues, there is fatherly care taken of each of the students. A number of men reside in the admirable residential college attached to the hospital. These are brought more directly under the influence of the authorities; but whether his son lives in the college or outside, the parent may rest assured that Guy's will do its very best for him.

### St. Bartholomew's.

St. Bartholomew's, in Smithfield, is the oldest hospital in London; it is the wealthiest, and its medical school is the senior metropolitan school. These facts imply much with regard to the status of the school. Age implies prestige; and the money which has been freely expended has procured an exceptionally large staff of teachers and tutors, as well as every appliance which the fully equipped medical school needs—not to speak of the generous benefactions which have provided very many prizes and scholarships open for competition at every stage of the student's career. The aristocratic school, some people would call it, but this epithet must not be used in these days without explaining that it is used without offence. At any rate it was a very courteous reception that Dr. Shore, the Warden of the College, accorded to our representative. Dr. Shore, who is a specialist in medical education, has his work at his finger ends, and the account he gave of his school and its arrangements was full and interesting. The fees at Bart's are higher than at any other London school—the composition fee is 150 guineas. Nevertheless the number of students has always been well maintained; and the school has been able to attract a large proportion of Oxford and Cambridge, as well as of London University, men. To the suggestion that students no doubt got value for the high fees, Dr. Shore replied, "Oh, yes; we have at least twenty more teachers than any other school." The Warden has been made aware that there is an impression abroad that Bart's is overcrowded. This, he holds, is quite a mistake; there is room for a good many more students. At Bart's the great object has been to afford a thorough scientific education, and to this end no expense has been spared. Full courses are provided in every subject; the museums and laboratories of the school cannot be seen without admiration; and the pathological museum is one of the finest of its class in its world. To the arrangements for clinical instruction at the hospital no less care has been devoted. The appointments are numerous enough to give every student work, and there are special classes in all the chief special diseases. At Bart's there are so many teachers that classes are small, and each student has the careful individual attention of the demonstrators in the subjects he is studying. Moreover, the Warden makes it a point to get to know every one of the men and to keep himself informed as to how they are doing their work. Students are expected to go forward to their degree examinations as they become due; and if they do not do so it becomes necessary to know the reason why. The youth who is obstinate in laziness or misconduct is reported to his parents. The authorities of the school make intimation that the holding of the ticket is conditional on the student's work and conduct being satisfactory to the authorities of the hospital and to the medical officers, and when it becomes absolutely necessary they do not hesitate to declare the ticket forfeited and to rusticate the offender. In addition to seeing that the men work, Dr. Shore takes some care for their comfort and good behaviour outside the school. Students who have parents in London of course reside with them; those who come up from the country do one of three things; they enter the residential college, or they board with one of the members of the staff, or they go into lodgings. At the residential college there is direct supervision of the men, but the accommodation in it is limited. A certain number board and have the advantage of the assistance in their studies of the gentlemen with whom they take up their quarters; but the majority go into lodgings. Dr. Shore keeps a list of approved lodgings as to which he has made careful inquiries, and, especially in the case of young men coming to London for the first time, he strongly urges that this list should be consulted. To the social welfare of the students the various societies and clubs and the recreation grounds at Winchmore Hill contribute. One of the societies—the Abernethian—has historical associations of a most interesting kind. This society is on the eve of celebrating its centenary.

### St. Thomas's.

One of the best known of the architectural features of London is the hospital of St. Thomas, on the south side of the Thames, opposite the Houses of Parliament. It is a magnificent situation crowned by buildings that do honour to the taste as well as to the philanthropy of the metropolis. Site and surroundings are often large factors in the choice of a medical school; in respect of these things St. Thomas's is pre-eminent among the medical schools in London, and the school is associated with an hospital which is structurally one of the finest in the world, which from the time when it was opened until now has been regarded as the model hospital. For the medical school buildings the same claim—and in view of the recent extensions—not unfairly, has been made. These additions were opened in June last, and they consist of two wings, one of them devoted to pathological and biological laboratories, the other to the purposes of the students' club. Of these buildings St. Thomas's men may now well be proud. There are more extensive connected with medical schools in London but none more admirably arranged. It was the privilege of our representative to be shown over the school the other day by Mr. Makins, the Dean. It would be difficult to speak too highly of the dining-room and reading-room—spacious lofty halls most comfortably furnished—nor of the pathological laboratory which in arrangement and fitting up may fairly be regarded as ideal. St. Thomas's gives special attention to the scientific side of medical study, and the instruction provided in the subjects of the London University and kindred examinations is excellent, as the results show. Nor have the opportunities for instruction which the most modern and the best equipped of the London hospitals affords been neglected. The arrangement and construction of an hospital are of the first importance in regard to the well-being of the patients, but it would not be difficult to show that they have a real bearing upon the efficiency of the cilinical instruction also. Not only so, but to have been trained at an hospital like St. Thomas's is to have learned a respect for sanitary conditions which must remain with a man for life. At St. Thomas's good teaching is supplemented by good discipline. Everything is done to promote pleasant companionship and healthy recreation. It is undoubtedly true that the opening of the dining room and the reading-room, which have been referred to, is fact of real importance in regard to the moral welfare of the student. These rooms help to make him comfortable and happy, and to do this is to encourage him to work, to render him less liable to get into temptation. There is one feature of the discipline at St. Thomas's that deserves special attention. The school prospectus speaks of "composition," not of "perpetual" fees, and it contains the following announcement:—

It should be understood that although the composition fees are intended to cover unlimited attendance on lectures and hospital practice, yet if a student fail to pass the several professional examinations within periods deemed reasonable by the school authorities, he may be required to pay additional fees for attendance at practical courses and technical classes, or his rights as a student may be suspended or determined at any time by the School Committee, with the approval of the treasurer.

The meaning in practice of this announcement is that when a student omits to sit for his examinations, or, having sat, fails to pass, the authorities are in a position to exact further fees. And it has been often found that this is the most effectual way of bringing the young man to his senses. St. Thomas's, it will thus be seen, is in a fair way to abolish the perpetual fee. But although paltering with examinations is visited with special censure, the student has supervision from the very first. In obtaining lodgings or board every assistance is offered, and parents are counselled to avail themselves of this assistance. A tendency to laziness is soon discovered and reproved, at first gently, and then sternly; and should it prove that in entering upon the study of medicine a young man has mistaken his vocation, his parents are courteously advised to choose for him a career in which he is better fitted to excel.

University College.

At one stage in its career University College bore the name of the University of London. In a weak moment it gave up the name, but it still retains the University character, and the medical school is one of its faculties. The men who come forth from University College have a character for learning which a long list of distinguished teachers has imparted to the school. For original work it has a brilliant and well-deserved reputation, which is well maintained by those on whom the mantles of such men as Liston, Syme, and Carswell have fallen. The school has long laboured under the disadvantage of an hospital small and old-fashioned in construction. But this is about to be remedied. A beginning is shortly to be made with new buildings, and University College does not mean to stop until it has got a completely new hospital. The medical school buildings are extensive, and in the matter of laboratories exceptionally well furnished. But here, too, important additions are in contemplation. University College prides herself on the success with which she has devoted herself to higher scientific work. At a rough estimate two-thirds of her men prepare for the London University degrees, and the highest honours of the London examinations have been carried off by this school oftener than by any other. There can be no doubt that in the reorganisation of medical education in London, in connection with the London University that is to be, the admirable work in science that has been done by this school will have due recognition. The success that has been attained in higher work speaks not only of the character of the teaching but of the character of the students. The men who come to University College are, for the greater part, men who are anxious to work. The spur is seldom needed; all the same a careful supervision is maintained, and if a youth shows a disposition to go wrong, he is at once taken in hand. University College has a Dean, a Vice-Dean, and also a Sub-Dean. The Dean is Professor Victor Horsley who, albeit that he is still a young man, has won for himself a place in the very front rank of medical science. In regard to medical education, Professor Horsley has decided views, and some of these he was good enough to impart to our representative. On one matter he laid special stress—that of the State degree. Everyone knows that in this country there is quite a number of bodies entitled to confer degrees in medicine. This has not only bred confusion in the public mind as to the value of the various qualifications, but it has led, it is held, in some cases to a laxness in examination. Mr. Horsley would substitute for the qualifications of the licensing corporations a State degree in medicine. The examination should be uniform and conducted under the direction of the General Medical Council. There would be certain difficulties in bringing about this simplification, but, once accomplished, the gain to the public would be great. The State degree, which should carry the title of Doctor, would be a guarantee that a certain uniform standard has been attained. Higher qualifications would still be open to those who wished them, and were able to win them. Professor Horsley has given a good deal of attention to the case of the young man who comes up from the country to London to study medicine, and he is deeply impressed with the risks that attend the course usually followed. The youth is still a youth, and yet he is expected to comport himself as a man. He is shot up from the country, where he has had home life and home comforts, into lonely lodgings in London. He is without friends, without society. If he has had a good training he may survive this ordeal, but who can wonder that so many go under. Mr. Horsley's view—and it is worthy of the attention of parents—is that young men should invariably be sent to board with a medical man, or with a family where they can have the civilising influences of home life and female society. He strongly deprecates the practice of sending inexperienced youths from the country into lodgings; and as for the additional expense of board, this would be slight in comparison with the social and moral advantages obtained.

### King's College.

King's College has a constitution substantially the same as that of University College; it has several faculties, of which the medical is one. The medical school is housed in well-known buildings in the Strand, near Somerset House, from which the College Hospital in Portugal Street, Lincoln's Inn Fields, is distant by a walk of between five and ten minutes. Both school and hospital have a history that is interesting, in some respects remarkable. For science instruction in London King's College has done and is doing much; in regard to the sciences that belong specially to medicine its record is very creditable. It was one of the first schools in this country to recognise the importance of laboratory work; it was one of the first to make adequate provision for instruction in bacteriology. In its numerous and well equipped laboratories research work of no mean order has been done. Special attention has been given to hygiene and public health, and in this department a full course is provided. There is a large staff of tutors, who give personal instruction in almost all the subjects of study. The College Hospital has been somewhat handicapped for want of funds; but it is known to all the world as the place where much of the life-work of Sir Joseph Lister, who is still honorary consulting surgeon, has been done. It is well known—for the fact has been much before the public of late—that King's College is in a special sense identified with the Church of England; all its teachers, in medicine as in other faculties, are members of the Church. This is not the place to discuss whether this is an advantage or a disadvantage; but it concerns parents to know that not only has the college ecclesiastical relations, but also that a system of moral and religious discipline prevails, which in some respects is unique. This system is described in "the regulations affecting medical students." Without the special permission of the Principal no one who is under the age of sixteen is allowed to begin the study of medicine—a sensible regulation that might be copied with advantage. Each student before matriculation must produce a testimonial of good moral character—a provision that might be useful were it not that in these days certificates of character are often as unreliable as they are easy to obtain. All matriculated students of the first year are required to attend lectures that are delivered once a week by the Principal, the Rev. Dr. Wace. The character of these lectures may be inferred from the subject that will be dealt with during the coming session, viz., "The Christian Creed and its Evidences." The Principal, however, may grant exemption from attendance at these lectures. The most noteworthy feature of the system at King's College is that at the close of each term the Dean of the Medical Faculty prepares a report of the attendance, general character, and conduct of each student, which is forwarded to his parent or guardian, after being submitted to and signed by the Principal. Although at all the schools the parent may on application obtain information as to his son's industry or progress, King's College alone supplies reports, at stated intervals, as a matter of course. The preparation of these reports must cost much time and trouble; but without raising the question of whether the practice is one that should be adopted by other schools, it cannot be doubted that it meets the wishes of many parents.

### Middlesex.

It proved convenient after King's College to visit Middlesex. This hospital, which is in Mortimer Street, has for well-nigh a century and a-half supplied hospital accommodation for the large industrial population north and south of Oxford Street. The medical school is of later origin; it was established in 1835. The school buildings are in the rear of the hospital. These have been repeatedly enlarged, and are ample and up to date. hey include a well-appointed residential college, which accommodates about thirty students. The hospital itself, which was renovated internally last year, has 324 beds, of which 34 are set apart for cancer cases. At present a new operating theatre is being constructed, which in the matter of safeguards against sepsis will leave nothing to be desired. Middlesex is a thriving school, which has done much excellent work in an unpretentious way. In one respect the authorities have had the courage to take ground of their own. The school does not undertake to teach the purely science subjects that are necessary for the Preliminary Examination of the University of London. Students who contemplate this examination are referred for instruction in science to University College or King's College. On passing the examination they come back to Middlesex to pursue their strictly medical studies. In a word, Middlesex is content to be an excellent medical school; it does not profess to be a science school as well. This course has the advantage that it enables the school to give its greatest energies to medicine and surgery proper, and its justification lies in the fact that in the final examinations for the London M.B. the school has been well able to hold its own with other schools. At the same time it should be noted that chemistry and biology as required for an ordinary qualification and prescribed by the General Medical Council, Middlesex does teach. Another feature of this school is that special provision is made for the needs of dental students, many of whom from the neighbouring dental hospitals in Great Portland Street and Leicester Square take the general course for the L.D.S. at Middlesex. In the staff of the school several important changes have been made during the past year. In the absence from London of Dr. Sidney Coupland, the Dean, our representative was referred to Mr. A. Pearce Gould, many years connected with the staff, who has lately been appointed lecturer in practical surgery. Mr. Pearce Gould, in the course of an interview, was good enough to give frank expression to his views as to the moral conditions of student life in London. He finds that there is an exaggerated idea among parents as to what is within the power of teachers in this matter. A boy —who as likely as not has had a bad training—is sent to London to attend medical classes and to live alone in lodgings. At the end of a year or two he returns to his parents a dissipated wreck. The parents turn round and blame the boy's teachers; they should have looked better after him. This is quite unjust. What the school can do—and what every school is bound to do—is to see that the men work. This is indeed much, but it is not all. The conditions of life in London make it quite impossible that the teachers should look after the youths after school hours. Well meant attempts have been made to do this, but they have not been attended with invariable success. Much—everything in fact—depends upon the home training the boy has had. If this has been good he will survive even the ordeal of solitary life in London. All the same Mr. Pearce Gould admits that he would not send a son of his to live alone in London lodgings. If it is beyond the means of the parents to board their boy with a respectable family, they should arrange for his sharing rooms with a senior student of good character and working habits—a plan that often answers very well indeed. It is very important, Mr. Pearce Gould holds, that parents should be warned against influencing boys who have no inclination for medical study to become medical students. The youth who is without interest in the work to which he has been put is almost certain to go wrong.

### St. George's.

Who that has been to Hyde Park Corner has not noticed the somewhat imposing building that occupies the angle formed by the junction of Hyde Park with the Green Park? This is St. George's Hospital, and at the rear is the medical school. The hospital puts at the disposal of the sick poor of London 355 beds, and it has a convalescent hospital near Wimbledon, to which patients are regularly drafted, with 100 beds. The medical school block is well planned and commodious. It includes a fine museum, a well-stocked library, a reading-room, and a refreshment-room, where students may have their meals at moderate charges. The hospital and school have many associations with the honoured name of John Hunter. It was he who founded the medical school; it was in one of the rooms of the hospital that he died; in the museum several interesting relics of Hunter are preserved. Whatever be the reason—has the site anything to do with it?—St. George's school is much resorted to by the sons of wealthy men. University men in considerable proportion find their way to it, also students who aspire to the army and navy medical service. At the service examinations the school is well represented. A good all-round medical education is provided; but St. George's is especially strong on the clinical side. The students have exceptional opportunities for clinical observation. At many hospitals it is the rule that students, unless they be clerks or dressers, are only admitted to the wards when accompanied by one of the members of the staff. At St. George's the governors have sufficient confidence in the good sense and gentlemanly behaviour of their men to allow them access to the wards at all convenient hours. From the students' point of view the privilege is a great one, and to their credit, be it said, it has not been abused. The arrangements for clinical instruction are very complete, and in connection with the hospital appointments a system has been worked out which has yielded excellent results. The student who receives one of these appointments virtually holds office for two years, and during this term he is enabled to obtain a wide and varied insight in practical medicine, surgery, and obstetrics that must prove invaluable in after life as a practitioner. St. George's men bear a good character, but the authorities do not omit to keep a careful watch upon the individual. It is a rule of the school that no student is allowed to present himself for any professional examination unless he has at least a fair chance of passing it. Examinations which are compulsory are regularly held by the various teachers in order to test whether the student has duly profited by his studies, and unless a satisfactory appearance is made, permission to sit for the "professional" is withheld. Parents who desire it will be furnished by the Dean with periodic reports of their son's progress. The Medical School Committee reserves to itself the right of requiring the withdrawal from the school of any student whose conduct is discreditable, and also of doing this without returning any portion of the fees that have been paid. St. George's has no residential college, but good lodgings in the neighbourhood of the hospital can easily be obtained. A list of approved lodgings is kept; also a list of houses belonging to medical men and others where St. George's students are received as boarders.

### St. Mary's.

St. Mary's Hospital and Medical School are situated in the far West, close to Paddington Station, the London terminus of the Great Western Railway. But it must not be thought that because it is a West-end hospital the demands upon its accommodation are small. On the contrary, its position so near Paddington enables it to serve the needs of a large population and a wide district. To its site also it is due that a goodly number of the students at the school are from the West of England. St. Mary's medical school has made rapid strides in public favour. Of this the number of entries each year bears witness. When it was put to Mr. Field, the Dean of the school, how this had come about, he modestly excused himself from giving an explanation. But one of the reasons undoubtedly is, that the authorities of the school have done their best to make it efficient, and to keep pace with the times. These efforts have borne fruit in the excellent appearance the school has made at various examinations. A reputation has thus been earned which has attracted many. The school is fairly wealthy in scholarships, and some of these are open only to University men. The provision for the teaching of the sciences is very good, and in the departments of bacteriology and public health especially noteworthy. Special attention is given to tutorial instruction; there are tutors in all the subjects, including medicine, surgery, and midwifery. Much has been achieved, but the governors have set their hearts on carrying out a plan that will add greatly to the usefulness of the hospital and to the efficiency of the medical school. A new wing, to be named, in memory of the late Duke of Clarence, the Clarence Memorial Wing, is to be built, at an estimated cost of £100,000. It goes without saying that when this wing has been built the available accommodation will have been substantially increased. Not only will it provide 100 additional beds, making 381 in all, but it will contain lying-in wards and a residential college. St. Mary's will thus be the first of the metropolitan general hospitals to establish lying-in wards; and it is easy to understand of what importance these wards will be in connection with the practical study of obstetrics. The school has already a small residential college in Westbourne Terrace, where students are boarded at the rate of £75 for the academical year; but it will be much more satisfactory to have the college directly adjoining the school. It is certainly to be hoped that the money to effect these very desirable improvements will be early forthcoming. In regard to discipline there is no special feature to note. When it proves necessary, the idleness or misconduct of a student is reported to his parents, and should the offender prove incorrigible he is dealt with by the Medical School Committee. But the general success of the school may be taken as evidence that the authorities do not have much trouble on this head.

### Charing Cross.

Charing Cross is classed as one of the smaller metropolitan schools, but the hospital is so well known, the school so vigorous, and many of its teachers so distinguished, that it seems unjust to a thriving institution to speak of it as small. To be sure, the hospital has only 175 beds, nevertheless, in virtue of its central site, it fills a very important part in the hospital work of London, and could ill be spared. Its beginnings were small, but its record is one of steady growth from 1818 until now. The governors of the hospital have all along maintained a lively interest in the medical school, and their enterprise and generosity have done much towards making it what it is. The school buildings, which adjoin the hospital, and, indeed, are connected with it by a subway, are substantial and well-arranged. Space in the Charing Cross region is not over-plentiful, nevertheless every requisite of the medical school, from the pathological laboratory on the upper floor to the students' club-room in the basement, has found its place. Excellent teachers give instruction in all the subjects of medical study. There are special classes for those who mean to go forward to the University examinations. There is a department of public health, which is fully equipped. The arrangements at the hospital afford ample opportunities for the objective study of disease; with surgical cases in particular Charing Cross is always well provided. Classes are formed for the study of special branches of disease. Charing Cross men are made free of the adjoining Royal Westminster Ophthalmic Hospital, where clinical instruction is given daily. For the benefit of students who are about to present themselves for the final examinations special classes, which are free, are held in winter and summer. In Mr. Stanley Boyd Charing Cross possesses a Dean who is in thorough sympathy with the student mind, and who may be trusted to maintain a kindly and wise interest in the intellectual and moral welfare of the young men. Mr. Stanley Boyd wishes special attention to be drawn to the notice in the prospectus, that "parents desiring information concerning their son's progress should apply to the Dean." The teachers are most anxious that parents should take advantage of this invitation. A record of attendances is carefully kept, and the student who is absent without explanation from class or hospital is brought to book. Regular examinations, which are compulsory, are held in all the subjects, and thus it is difficult, if not impossible, for negligence to remain undetected. It is one of the rules of the school that conduct on a part of a student outside the hospital or school which is calculated to discredit hospital or school renders the student liable to suspension or expulsion; and the youth so suspended or expelled has no claim for the return of his fees. With regard to the diligence and conduct of all the students the Dean is careful to keep himself informed. Deans are not infallible; but Mr. Stanley Boyd is not the man to undertake a duty to which he is not prepared to devote his best energies.

### Westminster.

The hospitals of London are somewhat unequally distributed. In this regard one of the most notable anomalies is that the district of which the House of Commons may be regarded as the centre has three general hospitals for its share, all within easy distance of each other—St. Thomas's, Charing Cross, and Westminster. Nevertheless, all three are so well filled that it cannot be said that the supply is greater than the demand. At any rate, those who are in charge of the Westminster Hospital have never found any difficulty in getting the 200 beds at their disposal occupied; indeed, the difficulty has been in the other direction. Like Charing Cross and St. Thomas's, Westminster has its medical school. The school, however, is of modern institution compared with the hospital, which is one of the oldest in London. The school buildings in Caxton Street are at some distance from the hospital, which adjoins the Abbey. Compared with those of Guy's and St. Bartholomew's these buildings are no doubt small. Nevertheless, of the space there is the best has been made, and there is no essential feature wherein they come short. It is an open secret that the teachers at Westminster, men of energy, many of them of eminence, have been doing their best to make the school thoroughly efficient. And they have been succeeding; witness the increase in the number of entries of late years; witness also the creditable appearance the school has made in the University of London and F.R.C.S. examinations. The Dean of Westminster—of the medical school, not the Abbey—Dr. Spencer, whose devotion to the school and pride in what has been achieved are unmistakable, in conversation with our representative dwelt upon one or two of its salient characters. Admittedly this is a small school, but this is in several respects an advantage. For one thing, it ensures more intimate personal relations between the teacher and student. At a large school, it is a necessity of the case that a large share of the work of supervision should be referred to demonstrators. The conditions at such a school as Westminster allow of the teacher himself doing work that is elsewhere done by proxy. The professor gets to know his men, and to know all about them. Is it on this account that at Westminster serious breaches of discipline are exceedingly rare? At hospital—provided the hospital is of fair size—the student at a small school has better opportunities for clinical observation; and it is worthy of note that there are some excellent clinical teachers at Westminister. The case for the small schools deserves to be stated, and whatever view one may take, the facts place it beyond dispute that at Westminster a thorough and complete medical education is provided, along with the personal supervision of competent and kindly teachers. In regard to the home life of the student, Dr. Spencer was not disposed to be too severe upon lodgings. He believes that life in lodgings trains young men to self-reliance and independence. The young men themselves prefer lodgings, and are apt to find the restrictions of the boarding-house irksome. On the other hand, there are cases of youths for whom it is important that they should be placed with a family where there they will be looked after.

### The School of Medicine for Women.

Among metropolitan medical schools this occupies a distinct position of its own as the only one at which women may obtain a complete medical education. The school is the outcome of a movement which has had to meet much opposition, and has had strong prejudices to overcome; courage and perseverance have, however, won the day—the lady doctor is now an accepted, in many quarters a welcome, fact. The oldest, and still the largest, School of Medicine for Women in this country, this school was opened in the present building in what is now Handel Street, Brunswick Square, so long ago as 1874. The first three years of its history were years of struggle; but the worst was over when the school had been recognised by one of the licensing bodies—the King and Queen's College of Physicians, Dublin—and when in 1877 lady students were admitted to the practice of the Royal Free Hospital, Gray's Inn Road. Since 1877 progress has been uninterrupted. There are now seven bodies, including the Universities of London, Ireland, Glasgow, and St. Andrew's, which confer degrees in medicine upon women. The London school opened in 1874 with twenty-three students; the number in attendance last session was 162. The last balance-sheet shows not only careful management, but a satisfactory sum on the right side. The school buildings meet all requirements, and, as those who have visited them can testify, they wear an aspect of neatness and homeliness that are sometimes not too conspicuous in the medical schools where man is supreme. Nevertheless, the friends of the school have set their hearts upon erecting new buildings, and the project is one that deserves all sympathy and support. It was expedient when the school was founded to appoint lecturers who held office in other medical schools in London, and the staff is still largely composed of gentlemen who also occupy important teaching posts in connection with one or other of the "male" metropolitan schools. Vacancies, however, have occurred, and, very properly, these have been filled by the appointment for the most part of ladies. And it is worthy of remark that quite recently the Governors of the Royal Free Hospital have appointed two assistant anæsthetists from among the lady graduates. The Dean of the school is Mrs. Garett Anderson, M.D., herself one of the pioneers in the movement, and the honorary secretary is Mrs. Thorne, one of the founders of the school, whose efforts in its behalf have been unwearying. Of the quality of the teaching and also of the intellectual energy of the students there is evidence in the fact that last year two ladies took the M.D. of London and one the M.D. of the Royal University of Ireland, in the appearances made at the examinations of the Society of Apothecaries and the conjoint Colleges of Scotland, and in the percentages of marks gained at the various class examinations for prizes and certificates. Special stress is laid upon obstetrics, in which Mrs. Scharlieb, M.D., is lecturer. As for clinical work there is a very thorough system under which every student has ample experience in clerking and dressing. At present the Royal Free Hospital is undergoing much needed additions and alterations which will soon be completed. During the past year a number of important appointments have been conferred upon lady doctors who have had their training at this school. The most notable incident in this regard is the appointment by the Secretary of State for India of Miss Annette Benson, M.D. (Lond.), to be senior physician to the Kawa Hospital, Bombay. Of Dr. Benson, whose career has been distinguished throughout, her *alma mater*, with good reason, is very proud.

### Fees.

On the subject of fees little has been said—not because it is unimportant, but because it is most convenient to deal with it by itself. At all the schools there is a "composition" or "perpetual" fee which covers all the classes necessary for an ordinary qualification as well as hospital practice—for special classes an extra fee being charged. This composition fee being taken as a basis, as may fairly be done, the ascending scale is as follows:—Westminster, £115; Charing Cross, £115 10s.; School for Women, £125; London, £126; Middlesex, £126; St. Mary's, £130; King's College, £135; University College, £141 10s.; St. George's, £145; St. Thomas's, £150; Guy's, £150; St. Bartholomew's, £157 10s.

Nor has any attempt been made to estimate the relative wealth in scholarships. It would have been of little profit to have done so; it must suffice, therefore, to refer those who desire information on this head to the prospectuses of the several schools, for which application should be made to the Deans or Medical School Secretaries.

So much for the medical schools of London. An attempt has been made to point out some of the distinctive features of each. And yet it must be confessed that few of the points wherein they differ are of great importance. It is because they have so many good things in common that it is so difficult to give one the preference over the other.

These hospital schools are the chief, but they are by no means the only agencies for medical education in London. Mr. Thomas Cooke's School of Anatomy and Physiology, at 40, Brunswick Square, deserves special mention, in view of the great success which as a tutor Mr. Cooke has had. His classes are recognised by the University of London and other licensing bodies. There are, moreover, numerous hospitals and asylums which wisely make available the opportunities they afford for the study of special forms of diseases. There are post-graduate lectures and demonstrations, and there are courses of instruction for the specialist. These, however, concern those who are sufficiently advanced to profit by them; and it is not from the point of view of the senior student or the graduate, but from that of the beginner, that "Medical Education in London" has been dealt with here.

